# The Expression of miR-211-5p in Sentinel Lymph Node Metastases of Malignant Melanoma Is a Potential Marker for Poor Prognosis †

**DOI:** 10.3390/ijms251910859

**Published:** 2024-10-09

**Authors:** Rose Kathrin Caroline Moritz, Nicole Ebelt, Tina Rattay, Jovine Ehrenreich, Cord Sunderkötter, Dennis Gerloff

**Affiliations:** 1 Department of Dermatology and Venereology, University Hospital Halle (Saale), Martin-Luther-University Halle-Wittenberg, 06120 Halle (Saale), Germany; rose.moritz@charite.de (R.K.C.M.); nicole.ebelt@yahoo.com (N.E.); tina.rattay@gmx.de (T.R.); jovine.ehrenreich@uk-halle.de (J.E.); cord.sunderkoetter@uk-halle.de (C.S.); 2 Department of Dermatology, Venereology and Allergology, Charité—Universitätsmedizin Berlin, Corporate Member of Freie Universität Berlin and Humboldt—Universität zu Berlin, 10117 Berlin, Germany

**Keywords:** miR-211-5p, malignant melanoma, biomarker, miRNA

## Abstract

Metastatic primary cutaneous melanoma is a frequently fatal disease despite recent therapeutic advances. Biomarkers to stratify patients’ prognosis are lacking. MicroRNAs (miRNAs) are small, non-coding RNAs. We aimed to determine the expression of miR-211-5p in primary tumors and metastases of malignant melanoma and its potential use as a prognostic biomarker. We performed in situ hybridization for miRNA-211-5p on 109 FFPE melanoma samples from 76 patients, including 31 paired primary tumor/metastasis samples. For validation, we performed in silico analyses of TCGA skin cutaneous melanoma (SKCM) cohort. High miR-211-5p expression was more frequent in primary tumors (70.8%) compared to metastases (39.3%). In metastases, it was associated with a significantly worse overall survival. Data from TCGA SKCM cohort confirmed that high miR-211-5p expression in melanoma metastases, but not primary tumors, is associated with worse overall survival. MiR-211-5p expression in metastases is associated with a shorter survival, emphasizing the potential of miR-211-5p as a risk predictor for a less favorable clinical outcome in metastatic disease. In situ hybridization could be implemented in a routine laboratory workflow and can be performed on diagnostic tissue.

## 1. Introduction

Primary cutaneous melanoma is responsible for 90% of deaths associated with skin cancer [1]. Its increasing incidence raises the need for further improvement in diagnostics and therapy. Novel biomarkers may help to predict prognosis more accurately and consequently optimize the selection of therapeutic options. MicroRNAs (miRNAs) are promising biomarker candidates for melanoma [2] and other tumors. For example, in diffuse large B-cell lymphoma, high serum expression levels of miR-21 are associated with improved relapse-free survival times [3]. In breast cancer, a signature of nine miRNAs was identified as a diagnostic marker [4]. MiRNAs are small, non-coding RNAs that regulate gene expression post-transcriptionally by directing the RNA-induced silencing complex (RISC) to complementary target sequences in the 3′-untranslated region (3′UTR) of messenger RNAs (mRNAs), resulting in the inhibition of the translation machinery or in the degradation of the targeted mRNA [5,6]. We demonstrated a pivotal role of miRNAs in the development and progression of melanoma [7,8]. The present study focuses on miR-211-5p. This miRNA is located within an intron of the MITF-regulated gene TRPM1, which is involved in melanin synthesis [9]. The characteristics of miR-211-5p in melanocytic tumors render it an intriguing biomarker candidate. TCGA data analysis showed a significantly poorer prognosis in melanoma with high expression of miR-211-5p, independent of other risk factors [10]. MiR-211-5p is highly expressed in differentiated melanocytes [11,12,13], but its expression is found to be decreased in dedifferentiated melanoma cells [11,12,13]. Previous in situ hybridization (ISH) analyses revealed decreased expression of miR-211-5p in melanomas compared to common and dysplastic nevi [14]. Overexpression of miR-211-5p in melanoma cells in vitro may either reduce proliferation, migration, and invasion [9,11,13] or result in the development of more aggressive tumors, increased proliferation of tumor cells, and resistance to BRAF/MEK inhibitors in vitro and in vivo [10,15,16]. This resistance is caused by the activation of the ERK5 pathway [16]. The oncogenic characteristics of miR-211-5p may be due to a modified immune function of the tumor microenvironment (TME) which is mediated by the target gene guanine nucleotide-binding protein subunit alpha-14 (GNA15). The inhibition of pyroptosis and augmented glycolysis have been described [10]. In addition, miR-211-5p is transported from melanocytes to various skin cells via melanosomes, leading to significant accumulation in, e.g., keratinocytes. The trafficking of miR-211-5p by melanosomes into dermal fibroblasts induces a carcinoma-associated fibroblast phenotype by targeting IGF2R, resulting in enhanced melanoma growth [17]. The transition of exosomal miR-211-5p from more aggressive tumor clones to their less aggressive surrounding melanoma cells could transfer their metastatic capacity in vitro [10]. The reason for the observed discrepancy in miR-211-5p function is not yet fully understood. It may be due to the expression of different sets of miR-211-5p target genes at different time points during melanoma progression or due to the use of various melanoma model systems. Regardless of the functional consequences of the induced overexpression of miR-211-5p, we are more interested in the natural expression of miRNA-211-5p in melanoma.

The aim of our study was to determine whether miR-211-5p is differentially expressed in primary tumors and metastases (using paired samples of the same individuals), and whether it might serve as a suitable prognostic biomarker in cutaneous melanoma.

To this end, we analyzed formalin-fixed paraffin-embedded (FFPE) patient-derived tumor samples by in situ hybridization, compared the expression of miR-211-5p in primary melanomas, soft tissue metastasis, and lymph node metastasis, and correlated it to the clinical data of the patients.

We observed a decrease in miR-211-5p expression from the primary tumor to sentinel lymph node metastases when comparing paired patient samples. However, patients with sustained expression of miR-211-5p in metastases showed a decrease in overall survival. We validated these results using in silico analyses of TCGA dataset.

## 2. Results

### 2.1. Patient Characteristics

We included 109 tissue samples from 48 primary melanomas (PTs), 51 sentinel lymph node metastases (SNMs), and 10 soft tissue metastases (STMs) from 76 patients (Table 1). The median age was 70.5 years (21–87); 31 patients were female (41%) and 45 were male (59%). The median follow-up time was 45 months (1-158 months). At the last follow-up, 35 patients were alive, 29 had died from melanoma, and 12 from other or unknown causes. For 24 out of 51 sentinel node metastases and 9 soft tissue metastases, the matched primary tumors from the same patient were evaluable. BRAF status was available in 11 patients; 6 of them had a BRAF V600 mutation. At the time of tissue resection, all patients were naïve to systemic therapy. During the course of their disease, 24 patients received adjuvant therapy with Interferon α. A total of 24 patients with metastatic disease received systemic therapy, including 11 with dacarbazine, 3 with temozolomide, and 5 with fotemustine. Five of the six patients with a documented BRAF mutation received BRAF inhibitor therapy. Four patients received checkpoint blockade therapy, three with PD1 monotherapy and one with ipilimumab and nivolumab.

### 2.2. miR-211-5p Is More Frequently Highly Expressed in Primary Melanomas

**Figure 1:**
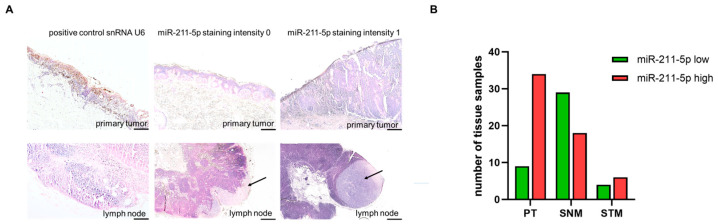
In situ hybridization analyses of miR-211-5p in patient tissue samples. (**A**) The images show the representative staining results from miR-211-5p in situ hybridization. U6, a ubiquitously expressed small nuclear RNA (snRNA), served as an internal positive control. The exemplary staining results of primary melanomas and lymph node metastases show a staining intensity grading system of 0 for low or non-expressed miR-211-5p compared to 1 for high expression. The arrows indicate melanoma metastases. The scale bars represent 100 µm. (**B**) The bars represent the absolute number of ISH analyses of tissue samples with low (green) or high (red) miR-211-5p expression.

To establish in situ hybridization, we stained normal healthy skin and benign nevi. We observed intensive staining for epidermal keratinocytes and melanocytes in the deep dermal component of benign melanocytic nevi with a congenital growth pattern, which complies with published data [14] (Supplementary Figure S1). Sufficient tissue for in situ hybridization was obtained from 43 of 48 primary tumors, 47 of 51 sentinel lymph node metastases, and all 10 soft tissue metastases. We found that high miR-211-5p expression was significantly more frequent in primary tumors (70.8%) as compared to sentinel lymph node metastases (35.3%, *p* < 0.01) (Figure 1B, Table 2). Soft tissue metastases showed increased expression of miR-211-5p in six (60%) out of ten analyzed tissue samples (Table 2, Figure 1B).

**Figure 2:**
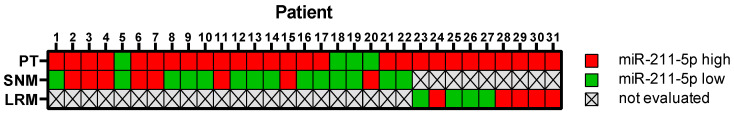
Evaluation of miR-211-5p expression in paired patient samples. Heatmap represents staining intensity for in situ hybridization of miR-211-5p in paired patient samples derived from primary tumors (PTs), sentinel lymph node metastases (SNMs), and soft tissue metastases (STMs). Red represents high miR-211-5p expression (staining intensity 1), while green indicates low or no miR-211-5p expression (staining intensity 0). Gray fields show unavailable samples.

Among the analyzed patient tissues, 31 primary tumors had paired metastatic specimens: 22 sentinel lymph node metastases and 9 soft tissue metastases. Overall, high miR-211-5p expression was found in 27 primary tumors (82%), 8 sentinel lymph node metastases (36%), and 5 soft tissue metastases (55%) (Figure 2). Out of the 22 paired samples of primary tumors and sentinel lymph node metastases, 7 pairs (32%) showed high expression in both. Meanwhile, nine pairs (41%) exhibited high expression of miR-211-5p in primary tumors, which decreased in the sentinel lymph node metastases. In three pairs (14%), we detected no expression of miR-211-5p, and in one pair (5%), miR-211-5p expression was low or absent in the primary tumor but increased in the paired sentinel lymph node metastasis (Figure 2).

The primary tumors of patients with available paired soft tissue metastases (*n* = 9) all showed high miRNA-211-5p expression, while their matched soft tissue metastases showed similarly high expression in five patients (55%), but low or no expression in four patients (44%) (Figure 2).

The results suggest that miR-211-5p expression varies among patients, but generally decreases with the progression of the disease.

### 2.3. High miR-211-5p Expression in Sentinel Lymph Node Metastasis Is Associated with Worse Patient Outcome

**Figure 3:**
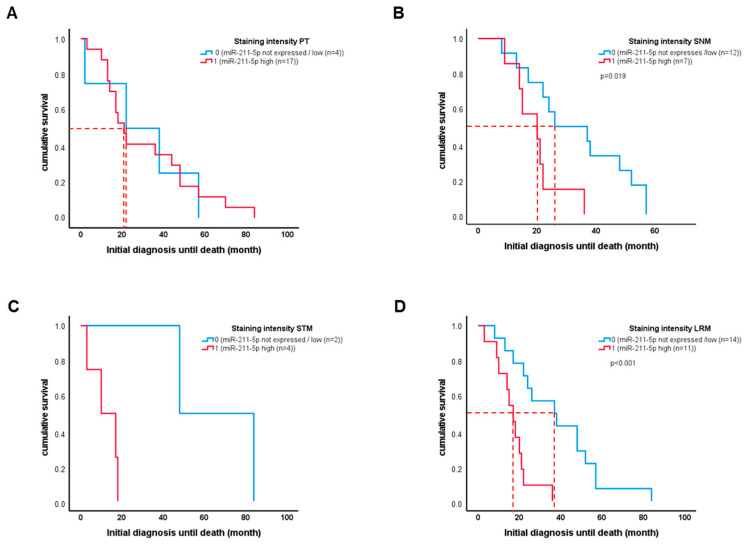
Graphs show survival probabilities dependent on miR-211-5p staining intensity (blue: 0 = no expression/low; red: 1 = high expression) for (**A**) primary melanoma samples (PTs), (**B**) sentinel lymph node metastases (SNMs), (**C**) soft tissue metastases (STMs), and (**D**) local regional metastases (LRMs) including SNMs and STMs.

Our analyses of the association between miR-211-5p expression and patient overall survival revealed no general correlation between high or low expression and survival (Figure 3A). However, high miR-211-5p expression in sentinel lymph node metastases was associated with a shorter survival in the subgroup of patients who died from metastatic disease (*n* = 29, *p* = 0.019) (Figure 3B). Although not statistically significant, patients with high miR-211-5p expression in soft tissue metastases tended to have shorter survival (Figure 3C).

When we combined patients with sentinel lymph node metastases and those with soft tissue metastases, we found that those with high expression of miR-211-5p revealed significantly worse survival (Figure 3D).

### 2.4. In Silico Data Analysis

**Figure 4:**
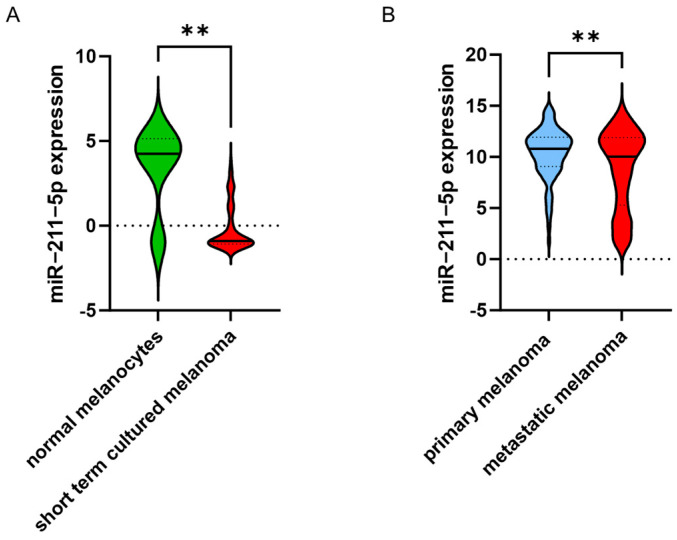
(**A**) miR-211-5p expression in normal melanocytes and patient-derived short-term cultured melanoma cells (data: Lin et al., 2008). (**B**) miR-211-5p expression in normal tissue and primary and metastatic tumors (TCGA) (** *p* ≤ 0.01).

To validate the results of our miR-211-5p ISH analyses, we examined additional publicly available datasets. We found that miR-211-5p is expressed significantly more highly in melanocytes than in short-term cultured primary melanoma cells (Figure 3A). A comparison of primary tumor samples and metastatic tumors showed significantly higher miR-211-5p expression in primary melanoma tumors (Figure 4B).

**Figure 5:**
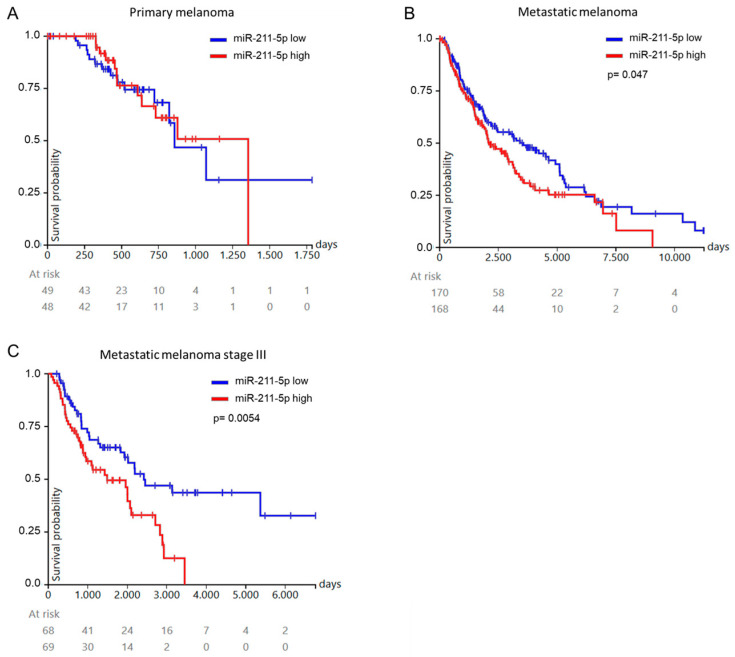
(**A**) High miR-211-5p expression is associated with poor survival of patients with metastatic melanoma. Kaplan–Meier curves show overall survival probability according to miR-211-5p expression in (**A**) TCGA primary melanoma patients, (**B**) TCGA metastatic melanoma patients, or (**C**) TCGA stage III metastatic melanoma patients.

To confirm miR-211-5p as a discriminator for poor prognosis in metastatic melanoma, we examined the relationship between miR-211-5p expression and overall patient survival in the skin cutaneous melanoma (SKCM) dataset of TCGA database. While in primary tumor samples high miR-211-5p expression was not related with shorter patient overall survival (Figure 5A), we found that high miR-211-5p expression in metastatic melanoma samples was associated with significantly (*p* = 0.047) poorer overall survival of patients (Figure 5B). In particular, when analyzing patients with stage III melanoma, high expression of miR-211-5p was even more significantly (*p* = 0.0054) correlated with reduced overall survival (Figure 5C).

## 3. Discussion

The objective of our study was to determine whether there is a difference in the expression of miR-211-5p between primary melanoma and metastases, and whether its expression as determined by in situ hybridization on FFPE tumor material might serve as a suitable predictive biomarker for melanoma patient outcomes.

In primary tumors, high expression of miR-211-5p was significantly more frequent than in sentinel lymph node metastases and was not linked to poorer survival. In contrast, in sentinel lymph node metastases and soft tissue metastases, high miR-211-5p expression was linked to significantly poorer overall patient survival. As this result was unexpected, we validated the results of our ISH analyses on further subsets of TCGA melanoma skin cancer dataset. The findings indicate that high expression of miR-211-5p in melanoma metastases, but not in primary tumors, is associated with unfavorable patient outcomes.

MiR-211-5p has been proposed as a potential discriminator between melanomas and melanocytic nevi, as it is highly expressed in normal, differentiated melanocytes and is less expressed in 90% of melanomas [14]. In our study, the majority of primary melanoma cells exhibited clear expression of miR-211-5p, despite a notable decrease in the expression of miR-211-5p in primary melanoma tumor cells in comparison to normal melanocytes in healthy skin and nevus melanocytes.

The expression of miR-211-5p was significantly more frequent in primary tumors as compared to sentinel lymph node metastases. Its expression decreased in the majority of matched paired samples from primary tumors to metastases. Interestingly, the subgroup of patients with high miR-211-5p expression in their sentinel lymph node metastases or soft tissue metastases had significantly shorter survival. A negative impact of high expression of miR-211-5p on patient survival was previously shown in an analysis of TCGA data by Zheng et al. [10]. To further validate these results, an in silico analysis was conducted on additional subgroups of TCGA dataset. This analysis confirmed that patients with metastatic melanoma had shorter survival when miR-211-5p expression was high, while no correlation between miR-211-5p expression in primary melanoma and overall survival of patients could be identified.

A loss of miR-211-5p is thought to be associated with the dedifferentiation of melanocytes and the initiation of melanoma. This assumption is based on the fact that miR-211-5p is highly expressed in normal melanocytes, and its expression is mostly decreased during melanoma initiation [13,14,18]. However, our results suggest that preserved expression of miR-211-5p in metastases is associated with worse patient outcomes. Paradoxically, this suggests that high miR-211-5p expression may also contribute to melanoma progression.

Among many potential targets of miR-211, some were previously identified to be of relevance in melanocyte pathology. Melanoma cells communicate with fibroblasts via extracellular vesicles that are released into the dermis prior to melanoma cell invasion. We have recently revealed that these vesicles contain, e.g., miR-92b-3p which contributes to the reprogramming of primary fibroblasts into cancer-associated fibroblasts (CAFs) by mediating the downregulation of PTEN [19]. CAFs facilitate the progression from primary to metastatic melanoma and promote proliferation, which is associated with reduced levels of IGF2R (insulin growth factor 2 receptor). Interestingly, IGF2R mRNA has been identified as a direct target of miR-211. In addition, putative target genes of miR-211 such as PPARGC1A, RRM2, and TAOK1 are highly upregulated in Vitiligo, which is a pigmentation disorder. A dysfunctional respiratory response of Vitiligo melanocytes could be reversed by miR211 overexpression, indicating that miR211 expression might also facilitate metabolic processes in melanoma cells [20].

The function of miR-211-5p is closely related to the function of its transcription factor MITF. MITF and miR-211-5p show a high correlation in their expression in melanoma in TCGA dataset (Supplementary Figure S2). Similar to miR-211-5p, MITF is also highly expressed in normal melanocytes and has an important function in melanocyte differentiation, whereas in melanoma, similar to miR-211-5p, variable expression of MITF was shown [21,22]. Interestingly, in metastatic melanoma, MITF amplification was associated with a decrease in patient survival [23], similar to our data for miR-211-5p.

Based on the correlation between MITF expression and its related function in melanoma, miR-211-5p may represent a surrogate marker for MITF. The association between miR-211-5p and MITF may explain the poorer survival of patients with metastatic melanoma when both are strongly expressed. Thus, in melanoma, MITF promotes proliferation, suppresses senescence, and is inversely correlated with immune infiltration [24].

We suggest that the effect of miRNA-211-5p overexpression in primary cutaneous melanoma and metastatic disease should be differentiated. The effect of the uptake of exosomal miRNA-211-5p by keratinocytes has not been studied so far, but among other significant changes in the tumor microenvironment, it could be essential for tumor control in early melanoma disease and melanocytic nevi.

Also, in other cancer entities, the effect of miR-211-5p cannot be explained by one-directional effects. In colorectal cancer [25] and head and neck cancer [26], miR-211-5p acts as an oncomiR, while it displays tumor-suppressive characteristics in pancreatic cancer [27] and ovarian cancer [28]. The seemingly ambivalent role of miR-211 is less surprising, considering the large number of potential targets and similar variable effects of other regulatory molecules, as recently reported for m^6^A RNA modification regulators [29].

Our results are based on retrospective analyses of patient tissue and in silico data. One advantage of our approach is that the survival data of patients treated between 2004 and 2016 are largely unaltered by the currently broadly applied and highly efficient treatment options in the adjuvant and metastatic setting.

To strengthen our observations, prospective multicenter studies including large patient numbers will be helpful to further quantify the predictive value of miR-211. High expression of miRNA-211 in the tissue of sentinel node metastases could then become a criterion for the selection of more aggressive (neo-)adjuvant therapies and shorter staging intervals during follow-up. These analyses could be integrated into prospective interventional clinical trials. In addition, miRNA-211 and its target genes may represent potential targets for the treatment of metastatic melanoma. Deep miRNA sequencing combined with whole-transcriptome sequencing techniques have successfully explored miRNA regulatory networks and could be useful tools to further describe the functional role of miRNA-211 [30].

In summary, while miR-211-5p expression is more frequently lost in metastases as compared to primary melanoma, its preserved expression in metastases is associated with poorer overall survival of patients. Therefore, this study emphasizes the potential of miR-211-5p as a risk predictor for a less favorable clinical outcome in metastatic disease.

## 4. Materials and Methods

### 4.1. Patient Samples

We retrospectively investigated the expression of miR-211-5p in 76 melanoma patients by in situ hybridization (ISH) on formalin-fixed and paraffin-embedded (FFPE) tissue samples from primary tumors (PTs), sentinel node metastases (SNMs), and soft tissue metastases (STMs). FFPE tissue samples were obtained from patients treated in our institution between 2004 and 2016. Patient consent was waived for the retrospective analyses of excess diagnostic tissue.

### 4.2. In Situ Hybridization

In situ hybridization was performed using the miRCURY LNA miRNA ISH (FFPE) Kits (Qiagen, Hilden, Germany) according to the manufacturer’s instructions. To detect miR-211-5p expression, we used the LNA-miR-211-5p probe (Qiagen, Hilden, Germany) as the internal control. U6 small nuclear RNA (snRNA) was detected using the LNA-RNUB6 probe (Qiagen, Hilden, Germany). The staining was mostly homogenous among melanocytes within one lesion but differed in intensity between different lesions or samples. Therefore, we chose to evaluate and score semiquantitatively the mean staining intensity of melanocytes per sample instead of recording a percentage of melanocytes.

The intensity of staining was scored as ISH grade 0 or “low expression” for no-to-low stainability and 1 or “high expression” for strong-to-very strong stainability of miR-211-5p in melanoma tissue (Figure 1A). We then correlated the intensity of expression to tumor stage and overall and disease-specific survival.

### 4.3. In Silico Dataset Analyses

To validate our results, we analyzed miR-211-5p expression in an additional patient cohort. We performed in silico data analyses by using publicly available datasets from Lin et al. and the skin cutaneous melanoma miRNA expression dataset of The Cancer Genome Atlas (TCGA) consortium on primary tumors and metastases of cutaneous melanoma.

### 4.4. Statistical Analyses

Statistical data analyses were performed by using Kaplan–Meier survival graphs, the log rank test and Chi square test using SPSS 25 (IBM, Armonk, NY, USA), and GraphPad PRISM 10.0.2. (Dotmatics, Bishop’s Stortford, UK).

### 4.5. Ethics Approval

This study was registered at the German Clinical Trials Register (DRKS) with the study number DRKS00024417. It was approved by the ethics committee of the medical faculty of the Martin-Luther-University Halle-Wittenberg with the vote number 2019-156 and followed the principles stated in the Declaration of Helsinki.

## Figures and Tables

**Table 1:** Patient characteristics.

Number of patients (%)	76 (100)
Age at diagnosis (median (range))	70.5 (21–87)
Sex	Male: 45/Female: 31
Died of tumor (%)	29 (38.2)
Overall survival (month) from first diagnosis to follow-up (mean ± sd)	54.5 ± 40.5
Breslow Index (mm) (mean ± sd)	4.7 ± 4.2
BRAF status (%)	
Wildtype	5 (6.6)
BRAF V600	6 (7.9)
Unknown	65 (85.5)
TNM classification (%)	
T1	6 (7.9)
T2	15 (19.7)
T3	22 (28.9)
T4	14 (18.4)
Pathological stage AJCC 2009 at first diagnosis (%)	
IA	2 (2.6)
IB	1 (1.3)
IIA	3 (3.9)
IIB	0 (0)
IIC	0 (0)
IIIA	1 (1.3)
IIIB	32 (42.1)
IIIC	6 (7.9)
III (unknown)	10 (13.2)
IV	21 (27.6)

sd: standard deviation; AJCC: American Joint Committee on Cancer.

**Table 2:** Frequency of staining intensities in melanoma tissue. Staining intensity was graded with 0 for low staining intensity or no staining of tumor cells and with 1 for high staining intensity in tumor cells. ISH: in situ hybridization; n.e.: not evaluable. * ISH 1 was significantly more frequent in primary tumors as compared to SNMs (*p* < 0.01).

	Total (%)	ISH 0/Low (%)	ISH 1/High (%)	n.e. (%)
Tissue number	109 (100)			
Primary tumor (PT)	48 (44)	9 (18.8)	34 (70.8) *	5 (10.4)
Sentinel node metastases (SNMs)	51 (47)	29 (56.9)	18 (35.3) *	4 (7.8)
Soft tissue metastases (STMs)	10 (9)	4 (40.0)	6 (60.0)	0 (0)

## Data Availability

Non-patient-related data that support the findings of this study are available within the article and its Supplementary Materials. Supporting patient data are not available due to data protection policies.

## Supplementary Materials

The following supporting information can be downloaded at: https://www.mdpi.com/article/10.3390/ijms251910859/s1.
