# Supplementary figures and images for: The Expression of miR-211-5p in Sentinel Lymph Node Metastases of Malignant Melanoma Is a Potential Marker for Poor Prognosis
Source: Int J Mol Sci. 2024 Oct 9;25(19):10859. doi: 10.3390/ijms251910859 (PMC11477290; doi:10.3390/ijms251910859)

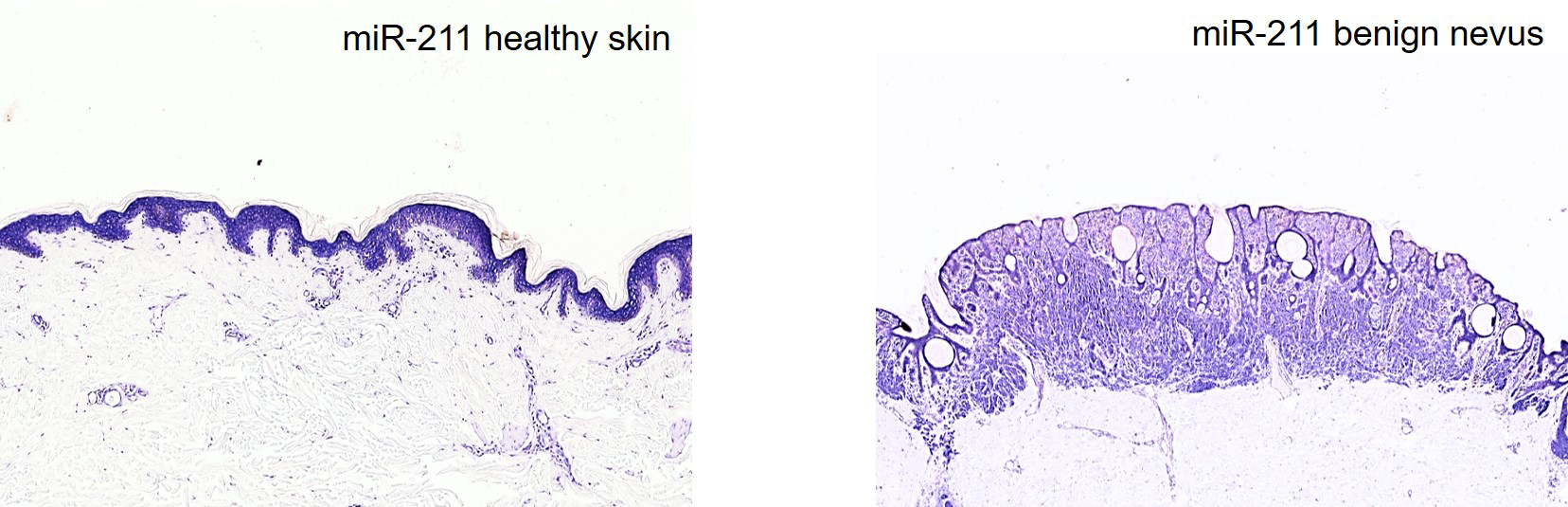

Supplement: Supplementary file 1 [file ijms-25-10859-s001.zip › supplementary Figure S1.tif]

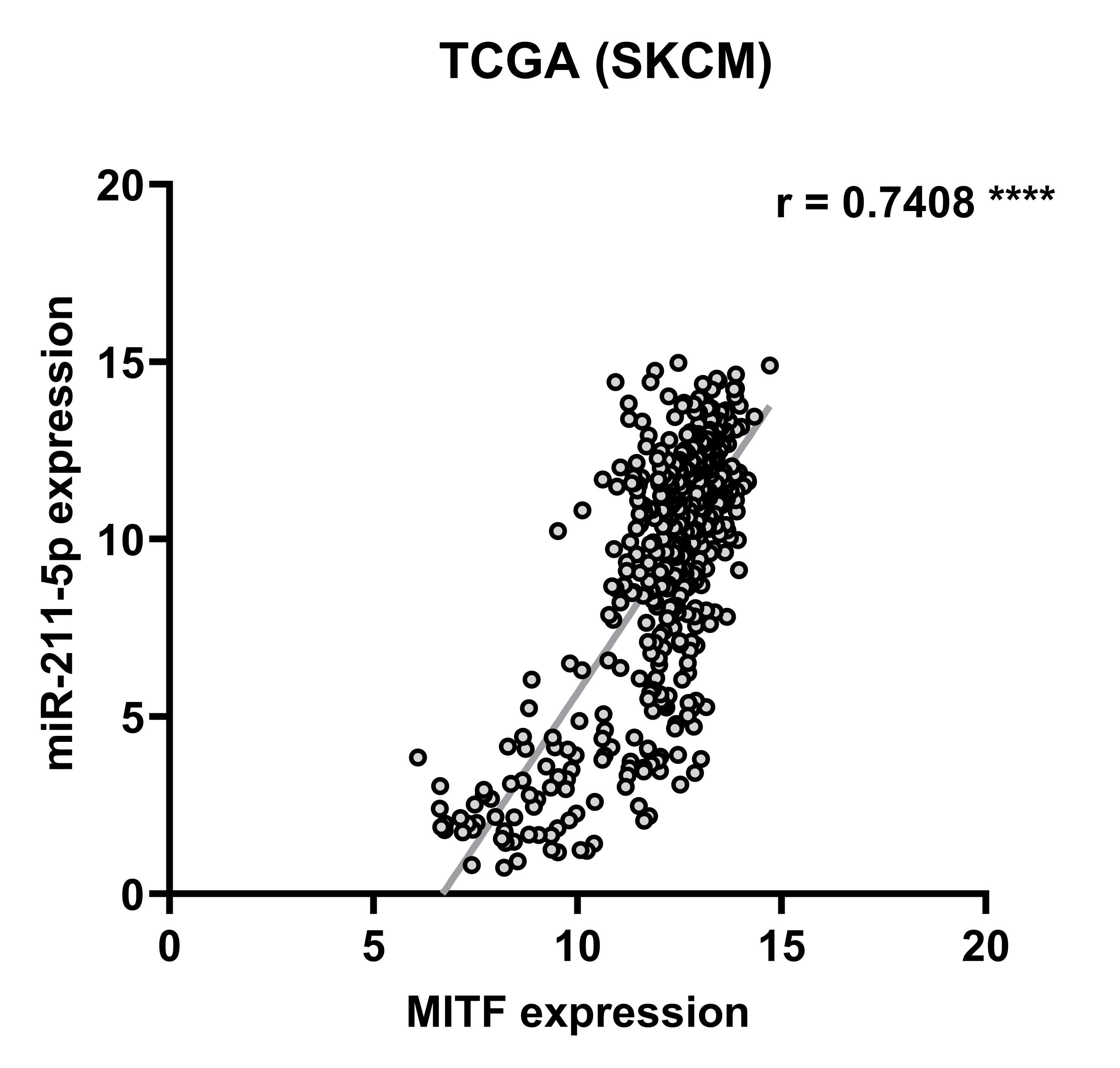

Supplement: Supplementary file 1 [file ijms-25-10859-s001.zip › supplementary Figure S2.tif]
